# 4-Ethynyl-*N*,*N*-di­phenyl­aniline

**DOI:** 10.1107/S1600536813029036

**Published:** 2013-10-31

**Authors:** Wan-Qiang Wang

**Affiliations:** a College of Chemical Engineering and Food Science, Hubei University of Arts and Science, Xiangyang 441053, People’s Republic of China

## Abstract

The asymmetric unit of the title compound, C_20_H_15_N, comprises two crystallographically independent mol­ecules (*A* and *B*). In each mol­ecule, the N atom adopts an approximately trigonal planar geometry, lying 0.009 (1) or 0.003 (1) Å from the plane defined by the C atoms of the aromatic substituents to which it is attached. In the crystal, mol­ecules are linked *via* C—H⋯π inter­actions, forming a three-dimensional structure.

## Related literature

For the synthesis and applications of the title compound, see: Onitsuka *et al.* (2006 ▶); Li *et al.* (2012 ▶). For the crystal structures of related compounds, see: Zhang *et al.* (2012 ▶); Narayanan *et al.* (2012 ▶).
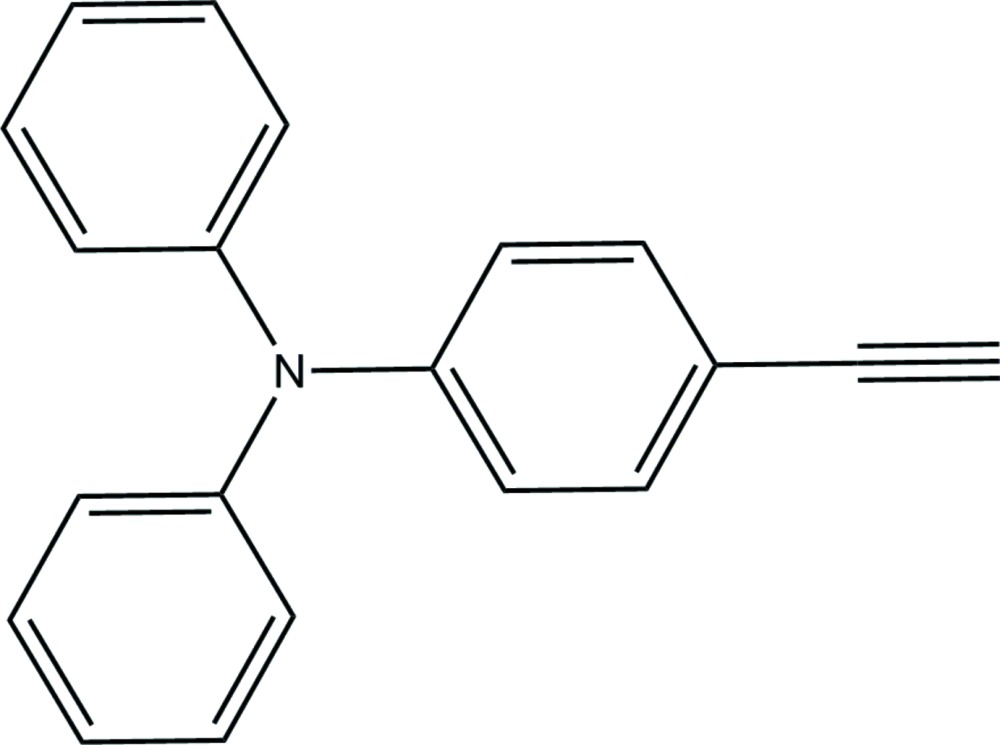


## Experimental

### 

#### Crystal data

C_20_H_15_N
*M* *_r_* = 269.33
Orthorhombic, 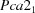

*a* = 9.0532 (8) Å
*b* = 16.8067 (15) Å
*c* = 19.2508 (18) Å
*V* = 2929.1 (5) Å^3^
*Z* = 8
Mo *K*α radiation
μ = 0.07 mm^−1^
*T* = 100 K
0.10 × 0.10 × 0.10 mm

#### Data collection

Bruker APEXII CCD diffractometer
Absorption correction: multi-scan (*SADABS*; Sheldrick, 1996 ▶) *T*_min_ = 0.993, *T*_max_ = 0.999
29115 measured reflections
8489 independent reflections
8095 reflections with *I* > 2σ(*I*)
*R*_int_ = 0.103

#### Refinement

*R*[*F* ^2^ > 2σ(*F* ^2^)] = 0.044
*wR*(*F* ^2^) = 0.121
*S* = 1.02
8489 reflections
379 parameters
1 restraint
H-atom parameters constrained
Δρ_max_ = 0.52 e Å^−3^
Δρ_min_ = −0.30 e Å^−3^

### 

Data collection: *APEX2* (Bruker, 2001 ▶); cell refinement: *SAINT* (Bruker, 2001 ▶); data reduction: *SAINT*; program(s) used to solve structure: *SHELXS97* (Sheldrick, 2008 ▶); program(s) used to refine structure: *SHELXL97* (Sheldrick, 2008 ▶); molecular graphics: *SHELXTL* (Sheldrick, 2008 ▶); software used to prepare material for publication: *SHELXTL*.

## Supplementary Material

Crystal structure: contains datablock(s) I, global. DOI: 10.1107/S1600536813029036/su2660sup1.cif

Structure factors: contains datablock(s) I. DOI: 10.1107/S1600536813029036/su2660Isup2.hkl

Supplementary material file. DOI: 10.1107/S1600536813029036/su2660Isup3.cml

Additional supplementary materials:  crystallographic information; 3D view; checkCIF report

## Figures and Tables

**Table 1:** Hydrogen-bond geometry (Å, °) *Cg*1, *Cg*3, *Cg*4, *Cg*5 and *Cg*6 are the centroids of the C1–C6, C15–C21, C21–C26, C29–C34 and C35–C40 rings, respectively.

*D*—H⋯*A*	*D*—H	H⋯*A*	*D*⋯*A*	*D*—H⋯*A*
C2—H2⋯*Cg*6	0.95	2.80	3.5375 (12)	136
C8—H8⋯*Cg*3^i^	0.95	2.66	3.5938 (15)	166
C12—H12⋯*Cg*5^ii^	0.95	2.77	3.6064 (15)	148
C20—H20⋯*Cg*4^iii^	0.95	2.72	3.5647 (12)	148
C28—H28⋯*Cg*5^iv^	0.95	2.92	3.5494 (17)	124
C34—H34⋯*Cg*1	0.95	2.88	3.6659 (13)	141

Symmetry codes: (i) 
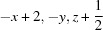
; (ii) 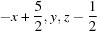; (iii) 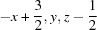; (iv) 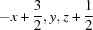.

**Fig. 1.**
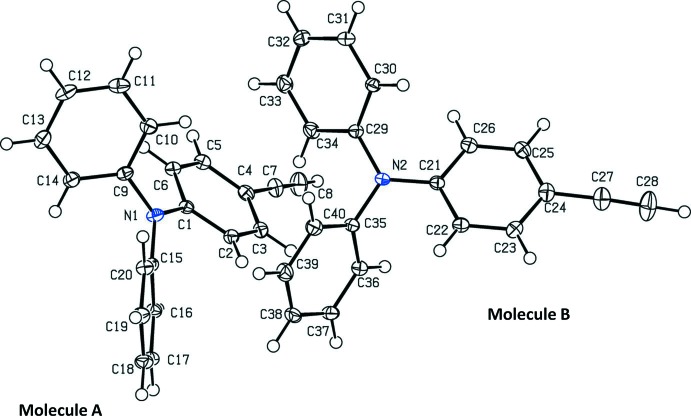
The molecular structure of the two independent molecules (A and B) of the title compound, with atom labelling. The displacement ellipsoids are drawn at the 30% probability level.

## supplementary crystallographic information

### 1. Comment

The title compound is an important intermediate in the synthesis of conjugated compounds used in materials chemistry (Li *et al.*, 2012). It has also been used as a bridging ligand in supramolecular chemistry (Onitsuka *et al.*, 2006). We herein report on its crystal structure.

The asymmetric unit of the title compound comprises two crystallographically independent molecules (A and B), as shown in Fig. 1. Its structure is analogous to that of 4-(2-Benzoylbenzoyl)-N,N-diphenylaniline (Narayanan *et al.*, 2012), and 4-(4-Nitrostyryl)-N,N-diphenylaniline (Zhang *et al.*, 2012). The distance between the two N atoms, N1 and N2, in the asymmetric unit is 5.973 (2) Å. The N atoms adopt approximate trigonal-planar geometry; in molecule A atom N1 lies 0.009 (1) Å out of the plane defined by atoms C1/C9/C15, while in molecule B atom N2 lies 0.003 (1) Å out of the plane defined by atoms C21/C29/C35.

In the crystal, molecules are linked by weak C—H···π interactions (Table 1) forming a three-dimensional structure.

### 2. Experimental

The title compound was synthesized according to the literature procedure (Onitsuka *et al.*, 2006). Yellow block-like crystals suitable for single-crystal X-ray diffraction analysis were grown by slow evaporation of a solution in hexane-ethanol (6:1).

### 3. Refinement

All the H atoms were positioned geometrically and refined as riding: C-H = 0.95 Å with *U*_iso_(H) = 1.2*U*_eq_(C).

### Crystal data

**Table:**

C_20_H_15_N	*F*(000) = 1136
*M**_r_* = 269.33	*D*_x_ = 1.221 Mg m^−^^3^
Orthorhombic, *P**c**a*2_1_	Mo *K*α radiation, λ = 0.71073 Å
Hall symbol: P 2c -2ac	Cell parameters from 9954 reflections
*a* = 9.0532 (8) Å	θ = 2.4–32.0°
*b* = 16.8067 (15) Å	µ = 0.07 mm^−^^1^
*c* = 19.2508 (18) Å	*T* = 100 K
*V* = 2929.1 (5) Å^3^	Block, yellow
*Z* = 8	0.10 × 0.10 × 0.10 mm

### Data collection

**Table:**

Bruker APEXII CCD diffractometer	8489 independent reflections
Radiation source: fine-focus sealed tube	8095 reflections with *I* > 2σ(*I*)
Graphite monochromator	*R*_int_ = 0.103
φ and ω scans	θ_max_ = 30.0°, θ_min_ = 1.2°
Absorption correction: multi-scan (*SADABS*; Sheldrick, 1996)	*h* = −12→12
*T*_min_ = 0.993, *T*_max_ = 0.999	*k* = −20→23
29115 measured reflections	*l* = −26→27

### Refinement

**Table:**

Refinement on *F*^2^	Primary atom site location: structure-invariant direct methods
Least-squares matrix: full	Secondary atom site location: difference Fourier map
*R*[*F*^2^ > 2σ(*F*^2^)] = 0.044	Hydrogen site location: inferred from neighbouring sites
*wR*(*F*^2^) = 0.121	H-atom parameters constrained
*S* = 1.02	*w* = 1/[σ^2^(*F*_o_^2^) + (0.0869*P*)^2^ + 0.0915*P*] where *P* = (*F*_o_^2^ + 2*F*_c_^2^)/3
8489 reflections	(Δ/σ)_max_ = 0.001
379 parameters	Δρ_max_ = 0.52 e Å^−^^3^
1 restraint	Δρ_min_ = −0.30 e Å^−^^3^

### Special details

**Table:**

Geometry. Bond distances, angles etc. have been calculated using the rounded fractional coordinates. All su's are estimated from the variances of the (full) variance-covariance matrix. The cell esds are taken into account in the estimation of distances, angles and torsion angles
Refinement. Refinement of *F*^2^ against ALL reflections. The weighted *R*-factor *wR* and goodness of fit *S* are based on *F*^2^, conventional *R*-factors *R* are based on *F*, with *F* set to zero for negative *F*^2^. The threshold expression of *F*^2^ > σ(*F*^2^) is used only for calculating *R*-factors(gt) *etc*. and is not relevant to the choice of reflections for refinement. *R*-factors based on *F*^2^ are statistically about twice as large as those based on *F*, and *R*- factors based on ALL data will be even larger.

### Fractional atomic coordinates and isotropic or equivalent isotropic displacement parameters (Å^2^)

**Table:**

	*x*	*y*	*z*	*U*_iso_*/*U*_eq_	
N1	0.97215 (10)	0.15515 (6)	0.18829 (5)	0.0151 (2)	
C1	1.01994 (11)	0.12696 (6)	0.25330 (6)	0.0131 (3)	
C2	0.92032 (11)	0.11708 (6)	0.30842 (6)	0.0154 (3)	
C3	0.96683 (12)	0.08555 (7)	0.37137 (6)	0.0161 (3)	
C4	1.11491 (12)	0.06340 (6)	0.38145 (6)	0.0161 (3)	
C5	1.21537 (12)	0.07606 (7)	0.32717 (6)	0.0161 (3)	
C6	1.16903 (11)	0.10742 (6)	0.26407 (6)	0.0145 (3)	
C7	1.16134 (13)	0.02747 (7)	0.44568 (6)	0.0195 (3)	
C8	1.19981 (16)	−0.00400 (9)	0.49830 (7)	0.0265 (3)	
C9	1.07362 (11)	0.19681 (7)	0.14450 (6)	0.0149 (3)	
C10	1.15056 (13)	0.26243 (7)	0.16999 (6)	0.0184 (3)	
C11	1.24849 (14)	0.30312 (7)	0.12685 (7)	0.0227 (3)	
C12	1.26630 (12)	0.27978 (8)	0.05795 (7)	0.0222 (3)	
C13	1.18980 (13)	0.21450 (8)	0.03265 (7)	0.0214 (3)	
C14	1.09451 (12)	0.17223 (7)	0.07592 (6)	0.0185 (3)	
C15	0.82610 (11)	0.14234 (6)	0.16355 (6)	0.0132 (3)	
C16	0.74983 (13)	0.07233 (6)	0.17946 (6)	0.0156 (3)	
C17	0.60599 (12)	0.06106 (7)	0.15513 (6)	0.0181 (3)	
C18	0.53783 (12)	0.11803 (8)	0.11378 (6)	0.0195 (3)	
C19	0.61546 (12)	0.18698 (7)	0.09708 (7)	0.0196 (3)	
C20	0.75783 (12)	0.19974 (6)	0.12192 (6)	0.0164 (3)	
N2	0.82287 (10)	0.34489 (6)	0.44105 (5)	0.0163 (3)	
C21	0.76463 (12)	0.37345 (6)	0.50402 (6)	0.0144 (3)	
C22	0.61135 (12)	0.38236 (7)	0.51185 (6)	0.0162 (3)	
C23	0.55195 (12)	0.40957 (7)	0.57389 (6)	0.0176 (3)	
C24	0.64336 (13)	0.42858 (7)	0.63045 (6)	0.0192 (3)	
C25	0.79684 (13)	0.42029 (7)	0.62215 (6)	0.0185 (3)	
C26	0.85630 (12)	0.39351 (7)	0.56026 (6)	0.0174 (3)	
C27	0.58063 (15)	0.45285 (8)	0.69544 (7)	0.0249 (3)	
C28	0.52423 (19)	0.47117 (11)	0.74931 (8)	0.0366 (4)	
C29	0.97123 (11)	0.35937 (6)	0.41982 (6)	0.0146 (3)	
C30	1.03888 (11)	0.43314 (7)	0.43085 (6)	0.0155 (3)	
C31	1.18418 (12)	0.44555 (7)	0.40952 (7)	0.0196 (3)	
C32	1.26298 (13)	0.38519 (8)	0.37653 (7)	0.0217 (3)	
C33	1.19565 (13)	0.31226 (8)	0.36518 (7)	0.0216 (3)	
C34	1.05057 (13)	0.29914 (7)	0.38670 (7)	0.0187 (3)	
C35	0.73086 (11)	0.29881 (7)	0.39602 (6)	0.0155 (3)	
C36	0.66127 (13)	0.23020 (7)	0.42044 (6)	0.0178 (3)	
C37	0.56952 (13)	0.18673 (7)	0.37672 (7)	0.0216 (3)	
C38	0.54757 (13)	0.21097 (8)	0.30816 (7)	0.0221 (3)	
C39	0.61849 (13)	0.27850 (8)	0.28360 (7)	0.0224 (3)	
C40	0.70958 (13)	0.32294 (7)	0.32706 (7)	0.0197 (3)	
H2	0.81990	0.13210	0.30260	0.0180*	
H3	0.89770	0.07890	0.40810	0.0190*	
H5	1.31650	0.06300	0.33360	0.0190*	
H6	1.23870	0.11570	0.22790	0.0170*	
H8	1.23040	−0.02900	0.54010	0.0320*	
H10	1.13630	0.27940	0.21660	0.0220*	
H11	1.30320	0.34690	0.14450	0.0270*	
H12	1.33090	0.30860	0.02830	0.0270*	
H13	1.20230	0.19850	−0.01430	0.0260*	
H14	1.04390	0.12680	0.05880	0.0220*	
H16	0.79590	0.03240	0.20680	0.0190*	
H17	0.55410	0.01380	0.16700	0.0220*	
H18	0.44010	0.11010	0.09720	0.0230*	
H19	0.57040	0.22590	0.06830	0.0240*	
H20	0.80870	0.24750	0.11060	0.0200*	
H22	0.54760	0.36960	0.47430	0.0190*	
H23	0.44800	0.41540	0.57820	0.0210*	
H25	0.86070	0.43330	0.65960	0.0220*	
H26	0.96040	0.38860	0.55570	0.0210*	
H28	0.47940	0.48570	0.79210	0.0440*	
H30	0.98570	0.47480	0.45290	0.0190*	
H31	1.22990	0.49560	0.41750	0.0230*	
H32	1.36190	0.39390	0.36200	0.0260*	
H33	1.24870	0.27090	0.34260	0.0260*	
H34	1.00550	0.24890	0.37880	0.0220*	
H36	0.67660	0.21320	0.46690	0.0210*	
H37	0.52150	0.14030	0.39360	0.0260*	
H38	0.48430	0.18140	0.27840	0.0270*	
H39	0.60480	0.29460	0.23670	0.0270*	
H40	0.75710	0.36940	0.31000	0.0240*	

### Atomic displacement parameters (Å^2^)

**Table:**

	*U*^11^	*U*^22^	*U*^33^	*U*^12^	*U*^13^	*U*^23^
N1	0.0120 (4)	0.0179 (4)	0.0153 (4)	−0.0025 (3)	−0.0018 (3)	0.0052 (3)
C1	0.0138 (4)	0.0115 (4)	0.0139 (5)	−0.0012 (3)	−0.0011 (3)	0.0007 (3)
C2	0.0141 (4)	0.0158 (5)	0.0162 (5)	−0.0006 (3)	−0.0002 (4)	−0.0007 (4)
C3	0.0178 (4)	0.0153 (4)	0.0151 (5)	0.0001 (4)	0.0003 (4)	−0.0005 (4)
C4	0.0194 (5)	0.0123 (4)	0.0167 (5)	−0.0002 (3)	−0.0025 (4)	0.0002 (4)
C5	0.0160 (4)	0.0148 (5)	0.0174 (5)	0.0001 (4)	−0.0023 (4)	0.0006 (4)
C6	0.0132 (4)	0.0136 (4)	0.0166 (5)	−0.0007 (4)	−0.0003 (3)	0.0010 (4)
C7	0.0234 (5)	0.0188 (5)	0.0163 (5)	0.0022 (4)	−0.0006 (4)	−0.0006 (4)
C8	0.0326 (6)	0.0287 (6)	0.0181 (5)	0.0082 (5)	−0.0011 (5)	0.0017 (5)
C9	0.0126 (4)	0.0158 (5)	0.0164 (5)	−0.0008 (4)	−0.0003 (4)	0.0039 (4)
C10	0.0203 (5)	0.0181 (5)	0.0167 (5)	−0.0030 (4)	−0.0020 (4)	0.0029 (4)
C11	0.0191 (5)	0.0214 (5)	0.0276 (6)	−0.0058 (4)	−0.0035 (4)	0.0063 (5)
C12	0.0137 (4)	0.0269 (6)	0.0259 (6)	−0.0021 (4)	0.0023 (4)	0.0101 (5)
C13	0.0182 (5)	0.0277 (6)	0.0184 (5)	0.0014 (4)	0.0034 (4)	0.0029 (5)
C14	0.0165 (4)	0.0197 (5)	0.0192 (5)	−0.0016 (4)	0.0013 (4)	0.0009 (4)
C15	0.0115 (4)	0.0148 (5)	0.0134 (5)	−0.0001 (3)	−0.0001 (3)	0.0003 (4)
C16	0.0158 (4)	0.0151 (5)	0.0159 (5)	−0.0011 (4)	−0.0005 (4)	0.0008 (4)
C17	0.0164 (5)	0.0189 (5)	0.0191 (5)	−0.0046 (4)	−0.0001 (4)	−0.0003 (4)
C18	0.0136 (4)	0.0235 (6)	0.0214 (6)	−0.0020 (4)	−0.0028 (4)	−0.0004 (4)
C19	0.0169 (4)	0.0207 (5)	0.0213 (5)	0.0015 (4)	−0.0025 (4)	0.0035 (4)
C20	0.0156 (4)	0.0150 (5)	0.0185 (5)	0.0001 (4)	−0.0010 (4)	0.0026 (4)
N2	0.0132 (4)	0.0187 (5)	0.0169 (4)	−0.0054 (3)	0.0007 (3)	−0.0042 (3)
C21	0.0160 (4)	0.0117 (4)	0.0155 (5)	−0.0017 (4)	0.0005 (4)	0.0001 (4)
C22	0.0153 (4)	0.0149 (5)	0.0183 (5)	−0.0006 (4)	−0.0010 (4)	0.0010 (4)
C23	0.0174 (4)	0.0152 (5)	0.0202 (5)	0.0019 (4)	0.0030 (4)	0.0025 (4)
C24	0.0242 (5)	0.0155 (5)	0.0179 (5)	0.0024 (4)	0.0016 (4)	0.0011 (4)
C25	0.0213 (5)	0.0174 (5)	0.0169 (5)	−0.0002 (4)	−0.0024 (4)	−0.0003 (4)
C26	0.0168 (4)	0.0172 (5)	0.0181 (5)	−0.0003 (4)	−0.0012 (4)	0.0000 (4)
C27	0.0292 (6)	0.0262 (6)	0.0193 (6)	0.0086 (5)	−0.0002 (5)	0.0010 (5)
C28	0.0411 (8)	0.0499 (9)	0.0187 (6)	0.0187 (7)	0.0034 (5)	0.0016 (6)
C29	0.0127 (4)	0.0155 (5)	0.0155 (5)	−0.0011 (3)	−0.0005 (3)	−0.0002 (4)
C30	0.0143 (4)	0.0139 (5)	0.0182 (5)	−0.0015 (3)	0.0000 (4)	−0.0024 (4)
C31	0.0158 (4)	0.0204 (5)	0.0226 (6)	−0.0050 (4)	0.0000 (4)	−0.0014 (4)
C32	0.0133 (4)	0.0304 (6)	0.0214 (6)	−0.0006 (4)	0.0005 (4)	−0.0038 (5)
C33	0.0187 (5)	0.0241 (6)	0.0221 (6)	0.0035 (4)	−0.0012 (4)	−0.0059 (4)
C34	0.0195 (5)	0.0155 (5)	0.0212 (5)	0.0010 (4)	−0.0011 (4)	−0.0039 (4)
C35	0.0133 (4)	0.0158 (5)	0.0173 (5)	−0.0024 (4)	−0.0004 (4)	−0.0030 (4)
C36	0.0178 (4)	0.0175 (5)	0.0182 (5)	−0.0029 (4)	0.0008 (4)	−0.0012 (4)
C37	0.0163 (4)	0.0206 (5)	0.0278 (6)	−0.0059 (4)	0.0031 (4)	−0.0064 (5)
C38	0.0139 (4)	0.0279 (6)	0.0246 (6)	−0.0021 (4)	−0.0017 (4)	−0.0103 (5)
C39	0.0208 (5)	0.0287 (6)	0.0176 (5)	0.0009 (4)	−0.0026 (4)	−0.0027 (5)
C40	0.0201 (5)	0.0206 (5)	0.0185 (5)	−0.0021 (4)	−0.0003 (4)	0.0000 (4)

### Geometric parameters (Å, º)

**Table:**

N1—C1	1.4064 (15)	C18—H18	0.9500
N1—C9	1.4300 (15)	C19—H19	0.9500
N1—C15	1.4218 (14)	C20—H20	0.9500
N2—C35	1.4301 (15)	C21—C22	1.4039 (15)
N2—C21	1.4064 (15)	C21—C26	1.4052 (16)
N2—C29	1.4249 (14)	C22—C23	1.3873 (16)
C1—C2	1.4025 (15)	C23—C24	1.4044 (16)
C1—C6	1.4045 (14)	C24—C25	1.4056 (17)
C2—C3	1.3880 (16)	C24—C27	1.4332 (18)
C3—C4	1.4048 (15)	C25—C26	1.3827 (16)
C4—C7	1.4388 (16)	C27—C28	1.196 (2)
C4—C5	1.4015 (16)	C29—C30	1.3991 (15)
C5—C6	1.3890 (16)	C29—C34	1.3954 (16)
C7—C8	1.1946 (18)	C30—C31	1.3937 (15)
C9—C14	1.3962 (16)	C31—C32	1.3933 (18)
C9—C10	1.3937 (16)	C32—C33	1.3862 (19)
C10—C11	1.3941 (17)	C33—C34	1.3948 (17)
C11—C12	1.3925 (19)	C35—C36	1.3956 (16)
C12—C13	1.3859 (18)	C35—C40	1.4014 (18)
C13—C14	1.3938 (17)	C36—C37	1.3900 (17)
C15—C20	1.3982 (15)	C37—C38	1.3955 (19)
C15—C16	1.3982 (15)	C38—C39	1.3871 (19)
C16—C17	1.3968 (16)	C39—C40	1.3921 (18)
C17—C18	1.3897 (17)	C22—H22	0.9500
C18—C19	1.3929 (17)	C23—H23	0.9500
C19—C20	1.3914 (16)	C25—H25	0.9500
C2—H2	0.9500	C26—H26	0.9500
C3—H3	0.9500	C28—H28	0.9500
C5—H5	0.9500	C30—H30	0.9500
C6—H6	0.9500	C31—H31	0.9500
C8—H8	0.9500	C32—H32	0.9500
C10—H10	0.9500	C33—H33	0.9500
C11—H11	0.9500	C34—H34	0.9500
C12—H12	0.9500	C36—H36	0.9500
C13—H13	0.9500	C37—H37	0.9500
C14—H14	0.9500	C38—H38	0.9500
C16—H16	0.9500	C39—H39	0.9500
C17—H17	0.9500	C40—H40	0.9500
			
C1—N1—C9	119.47 (9)	C20—C19—H19	120.00
C1—N1—C15	122.22 (9)	C15—C20—H20	120.00
C9—N1—C15	118.30 (9)	C19—C20—H20	120.00
C21—N2—C29	122.84 (9)	N2—C21—C22	119.97 (10)
C21—N2—C35	119.27 (9)	N2—C21—C26	121.64 (10)
C29—N2—C35	117.89 (9)	C22—C21—C26	118.39 (10)
N1—C1—C2	121.02 (9)	C21—C22—C23	120.71 (10)
N1—C1—C6	120.38 (10)	C22—C23—C24	120.93 (10)
C2—C1—C6	118.60 (10)	C23—C24—C25	118.15 (10)
C1—C2—C3	120.71 (9)	C23—C24—C27	120.53 (11)
C2—C3—C4	120.75 (10)	C25—C24—C27	121.28 (11)
C3—C4—C7	120.58 (10)	C24—C25—C26	121.00 (11)
C3—C4—C5	118.43 (10)	C21—C26—C25	120.80 (10)
C5—C4—C7	120.99 (10)	C24—C27—C28	177.63 (15)
C4—C5—C6	120.91 (10)	N2—C29—C30	121.36 (9)
C1—C6—C5	120.54 (10)	N2—C29—C34	119.50 (9)
C4—C7—C8	178.53 (13)	C30—C29—C34	119.14 (10)
C10—C9—C14	119.96 (10)	C29—C30—C31	120.07 (10)
N1—C9—C14	119.97 (10)	C30—C31—C32	120.57 (11)
N1—C9—C10	120.07 (10)	C31—C32—C33	119.37 (11)
C9—C10—C11	119.76 (11)	C32—C33—C34	120.47 (12)
C10—C11—C12	120.19 (11)	C29—C34—C33	120.38 (11)
C11—C12—C13	119.99 (12)	N2—C35—C36	120.41 (10)
C12—C13—C14	120.18 (12)	N2—C35—C40	119.84 (10)
C9—C14—C13	119.88 (11)	C36—C35—C40	119.75 (10)
C16—C15—C20	119.20 (10)	C35—C36—C37	120.01 (11)
N1—C15—C16	120.88 (10)	C36—C37—C38	120.29 (11)
N1—C15—C20	119.91 (9)	C37—C38—C39	119.69 (12)
C15—C16—C17	120.07 (10)	C38—C39—C40	120.56 (12)
C16—C17—C18	120.84 (10)	C35—C40—C39	119.70 (11)
C17—C18—C19	118.78 (10)	C21—C22—H22	120.00
C18—C19—C20	121.09 (11)	C23—C22—H22	120.00
C15—C20—C19	120.01 (10)	C22—C23—H23	120.00
C1—C2—H2	120.00	C24—C23—H23	120.00
C3—C2—H2	120.00	C24—C25—H25	120.00
C4—C3—H3	120.00	C26—C25—H25	119.00
C2—C3—H3	120.00	C21—C26—H26	120.00
C4—C5—H5	120.00	C25—C26—H26	120.00
C6—C5—H5	120.00	C27—C28—H28	180.00
C1—C6—H6	120.00	C29—C30—H30	120.00
C5—C6—H6	120.00	C31—C30—H30	120.00
C7—C8—H8	180.00	C30—C31—H31	120.00
C11—C10—H10	120.00	C32—C31—H31	120.00
C9—C10—H10	120.00	C31—C32—H32	120.00
C10—C11—H11	120.00	C33—C32—H32	120.00
C12—C11—H11	120.00	C32—C33—H33	120.00
C11—C12—H12	120.00	C34—C33—H33	120.00
C13—C12—H12	120.00	C29—C34—H34	120.00
C14—C13—H13	120.00	C33—C34—H34	120.00
C12—C13—H13	120.00	C35—C36—H36	120.00
C13—C14—H14	120.00	C37—C36—H36	120.00
C9—C14—H14	120.00	C36—C37—H37	120.00
C15—C16—H16	120.00	C38—C37—H37	120.00
C17—C16—H16	120.00	C37—C38—H38	120.00
C16—C17—H17	120.00	C39—C38—H38	120.00
C18—C17—H17	120.00	C38—C39—H39	120.00
C19—C18—H18	121.00	C40—C39—H39	120.00
C17—C18—H18	121.00	C35—C40—H40	120.00
C18—C19—H19	119.00	C39—C40—H40	120.00
			
C9—N1—C1—C2	152.80 (10)	C9—C10—C11—C12	1.86 (18)
C15—N1—C1—C2	−28.48 (15)	C10—C11—C12—C13	−1.89 (18)
C9—N1—C1—C6	−27.76 (15)	C11—C12—C13—C14	0.24 (18)
C15—N1—C1—C6	150.96 (10)	C12—C13—C14—C9	1.42 (18)
C1—N1—C15—C16	−34.25 (16)	N1—C15—C16—C17	179.51 (10)
C9—N1—C15—C16	144.49 (11)	N1—C15—C20—C19	179.36 (11)
C1—N1—C15—C20	146.66 (11)	C20—C15—C16—C17	−1.39 (17)
C1—N1—C9—C10	−53.62 (15)	C16—C15—C20—C19	0.25 (17)
C15—N1—C9—C10	127.61 (11)	C15—C16—C17—C18	1.37 (17)
C1—N1—C9—C14	127.04 (11)	C16—C17—C18—C19	−0.19 (17)
C15—N1—C9—C14	−51.73 (14)	C17—C18—C19—C20	−0.97 (18)
C9—N1—C15—C20	−34.61 (15)	C18—C19—C20—C15	0.94 (18)
C29—N2—C35—C40	−56.40 (14)	N2—C21—C22—C23	179.24 (10)
C21—N2—C29—C30	−39.77 (16)	N2—C21—C26—C25	−178.96 (11)
C29—N2—C21—C22	157.71 (10)	C22—C21—C26—C25	0.91 (17)
C21—N2—C35—C40	123.98 (11)	C26—C21—C22—C23	−0.63 (17)
C29—N2—C21—C26	−22.42 (16)	C21—C22—C23—C24	−0.25 (18)
C35—N2—C21—C26	157.17 (10)	C22—C23—C24—C27	−176.87 (12)
C21—N2—C35—C36	−55.59 (15)	C22—C23—C24—C25	0.84 (17)
C29—N2—C35—C36	124.03 (11)	C27—C24—C25—C26	177.14 (12)
C21—N2—C29—C34	140.89 (12)	C23—C24—C25—C26	−0.56 (17)
C35—N2—C29—C34	−38.72 (15)	C24—C25—C26—C21	−0.32 (18)
C35—N2—C21—C22	−22.70 (15)	N2—C29—C30—C31	180.00 (11)
C35—N2—C29—C30	140.63 (11)	C34—C29—C30—C31	−0.66 (17)
N1—C1—C6—C5	−176.97 (10)	N2—C29—C34—C33	179.65 (11)
C2—C1—C6—C5	2.48 (15)	C30—C29—C34—C33	0.29 (18)
C6—C1—C2—C3	−2.65 (15)	C29—C30—C31—C32	0.61 (19)
N1—C1—C2—C3	176.81 (10)	C30—C31—C32—C33	−0.2 (2)
C1—C2—C3—C4	0.49 (17)	C31—C32—C33—C34	−0.2 (2)
C2—C3—C4—C7	−177.22 (10)	C32—C33—C34—C29	0.1 (2)
C2—C3—C4—C5	1.85 (16)	N2—C35—C36—C37	178.62 (10)
C7—C4—C5—C6	177.05 (10)	C40—C35—C36—C37	−0.95 (17)
C3—C4—C5—C6	−2.01 (16)	N2—C35—C40—C39	−179.21 (10)
C4—C5—C6—C1	−0.16 (17)	C36—C35—C40—C39	0.37 (17)
C10—C9—C14—C13	−1.45 (17)	C35—C36—C37—C38	0.58 (18)
N1—C9—C14—C13	177.89 (10)	C36—C37—C38—C39	0.38 (18)
N1—C9—C10—C11	−179.53 (10)	C37—C38—C39—C40	−0.97 (19)
C14—C9—C10—C11	−0.19 (17)	C38—C39—C40—C35	0.60 (18)

### Hydrogen-bond geometry (Å, º)

Cg1, Cg3, Cg4, Cg5 and Cg6 are the centroids of the C1–C6, C15–C21, C21–C26, C29–C34 and C35–C40 rings, respectively.

**Table:**

*D*—H···*A*	*D*—H	H···*A*	*D*···*A*	*D*—H···*A*
C2—H2···*Cg*6	0.95	2.80	3.5375 (12)	136
C8—H8···*Cg*3^i^	0.95	2.66	3.5938 (15)	166
C12—H12···*Cg*5^ii^	0.95	2.77	3.6064 (15)	148
C20—H20···*Cg*4^iii^	0.95	2.72	3.5647 (12)	148
C28—H28···*Cg*5^iv^	0.95	2.92	3.5494 (17)	124
C34—H34···*Cg*1	0.95	2.88	3.6659 (13)	141

Symmetry codes: (i) −*x*+2, −*y*, *z*+1/2; (ii) −*x*+5/2, *y*, *z*−1/2; (iii) −*x*+3/2, *y*, *z*−1/2; (iv) −*x*+3/2, *y*, *z*+1/2.
